# Tolerance of pentose utilising yeast to hydrogen peroxide-induced oxidative stress

**DOI:** 10.1186/1756-0500-7-151

**Published:** 2014-03-17

**Authors:** Jennifer Spencer, Trevor G Phister, Katherine A Smart, Darren Greetham

**Affiliations:** 1 School of Biosciences, University of Nottingham, Loughborough, Leics LE12 5RD, UK

## Abstract

**Background:**

Bioethanol fermentations follow traditional beverage fermentations where the yeast is exposed to adverse conditions such as oxidative stress. Lignocellulosic bioethanol fermentations involve the conversion of pentose and hexose sugars into ethanol. Environmental stress conditions such as osmotic stress and ethanol stress may affect the fermentation performance; however, oxidative stress as a consequence of metabolic output can also occur. However, the effect of oxidative stress on yeast with pentose utilising capabilities has yet to be investigated.

**Results:**

Assaying for the effect of hydrogen peroxide-induced oxidative stress on *Candida*, *Pichia* and *Scheffersomyces* spp. has demonstrated that these yeast tolerate hydrogen peroxide-induced oxidative stress in a manner consistent with that demonstrated by *Saccharomyces cerevisiae. Pichia guillermondii* appears to be more tolerant to hydrogen peroxide-induced oxidative stress when compared to *Candida shehatae*, *Candida succiphila* or *Scheffersomyces stipitis*.

**Conclusions:**

Sensitivity to hydrogen peroxide-induced oxidative stress increased in the presence of minimal media; however, addition of amino acids and nucleobases was observed to increase tolerance. In particular adenine increased tolerance and methionine reduced tolerance to hydrogen peroxide-induced oxidative stress.

## Background

Yeasts may be confronted with a combination of different physical, biological, or chemical stresses during bio-ethanol fermentations. At the beginning of fermentation, yeast are subjected to osmotic stress due to high sugar levels. Yeast encounter osmotic shock when propagated into the wort, a complex media containing high concentrations of sugars [1]. Use of high gravity worts has been shown to reduce yeast viability, growth and fermentation profiles [2].

In addition, during fermentation other stress conditions become relevant such as ethanol accumulation, pH downshift, a shift to anaerobic growth and nutrient limitation [3,4]. During fermentation, accumulation of ethanol has been shown to inhibit yeast growth and viability [5-7].

Oxygen is an essential component of the brewing process and is required for the synthesis of unsaturated fatty acid and sterols [8]. The yeast cell is continuously exposed to oxygen during brewery propagation, a process whereby yeast cells are grown from stock cultures to a sufficient quantity for transfer into fermentation vessels. Oxygen is typically depleted within the first 12 hrs of fermentation which continues under anaerobic conditions.

Reactive oxygen species (ROS) free radicals such as superoxide anion (O_2_^−^), hydrogen peroxide (H_2_O_2_) and the hydroxyl radical (^.^HO) are generated as by-products of cellular metabolism [9]. Under some conditions ROS can disrupt a diverse array of biological processes [10]. ROS can damage a variety of cellular components, including DNA, proteins and unsaturated lipids [11]. ROS can damage the mitochondria causing the formation of respiratory-deficient petites [12-14]. ROS has been shown to have a direct role in cellular aging [15], replicant life-span has been related to anti-oxidant potential of the cell and the number of re-pitches into a fermentation [16,17]. Oxidative stress induced by free radical produced by aerobic metabolism may play a role in replicative aging of the yeast causing progressive deterioration of the cells as post fermentation harvesting potentially selects a higher proportion of aging yeast [18].

Toxicity arises from lipid peroxidation, production of alkoxyl/peroxyl radicals and aldehyde compounds, which are products of free radical lipid reactions triggered by ROS [19-21]. Yeast response to free radicals is diverse, enzymatic (thioredoxins, glutaredoxins, catalases, superoxide dismutasis) and non-enzymatic (glutathione, D-erythroascorbic acid, and trehalose) responses are employed by the yeast to overcome oxidative stress [22-26]. These responses differ during the course of fermentation for example, cellular superoxide dismutase and catalase activities rapidly decline during fermentation in semi-defined wort medium as the oxygen levels in the medium decline [27].

Lignocellulosic material is a cheap raw material for ethanol production and is composed of lignin, hemicelluloses, and cellulose. Cellulose is composed of long chains of glucose molecules and it is encapsulated by lignin. Conversion of cellulose derived monomeric sugars to ethanol has been shown in *S. cerevisiae* fermentations [28], however, this yeast is unable to utilise pentose sugars liberated from hemicellulose, the pentose polysaccharide component of plant cell walls without genetic modification [29].

*Candida, Pichia* and *Scheffersomyces* (previously named *Pichia stipitis*) currently represent the best natural yeast for efficient utilisation of pentose sugars into ethanol or other refined products [30]. Although much work has been done on the conversion of pentoses to ethanol, little is known about their response to the stresses posed by fermentation. Understanding the response of pentose utilising yeast to oxidative stress (induced by hydrogen peroxide in this study) will lead to an improvement in yeast strains for the production of ethanol from all available sugars post deconstruction of lignocellulosic material (LCM).

## Methods

### Yeast strains and growth conditions

*Saccharomyces cerevisiae* NCYC 2592*, Candida shehatae* NCYC 2389 and 3781, *Candida succiphila* NCYC 1403, *Pichia guillermondii* NCYC 441, 443 and 3063 and *Scheffersomyces stipitis* NCYC 1541 and 1542 were utilised in this study. All strains were obtained from the National Centre of Yeast collections (NCYC, Bristol, UK) and maintained on 10 g/L yeast extract, 20 g/L peptone, and 20 g/L glucose (YPD).

### Sensitivity to hydrogen peroxide-induced oxidative stress

Sensitivity to hydrogen peroxide-induced oxidative stress was determined by growing cells to stationary phase in YPD at 30°C. Optical density (OD_600_) was measured and adjusted to an OD_600_ of 1.0 corresponding to 10^7^ cells/ml. Cells were then serially ten-fold diluted and 5 μl was spotted onto YPD agar plates containing hydrogen peroxide (0-5 mM) as appropriate. Sensitivity to hydrogen peroxide was also measured using yeast nitrogen base (YNB) as a carbon source, as above, but the yeast were grown in 6% glucose, and 0.67% YNB, stressed and viable cells were counted after incubation on YPD plates.

### Viability assays

Viability was determined by growing cells in 50 mL YPD broth at 30°C until an OD_600_ reading of between 0.4 and 0.6 was reached indicating cells were at mid-exponential phase of growth. Cells were then treated with hydrogen peroxide at different concentrations for 15 minutes at 30°C, and shaken at 180 rpm in an orbital shaker, in an aerobic environment. After incubation, OD was re-assessed, cells were then diluted in fresh medium to a concentration of 1 × 10^4^ cells/mL and 10 μL plated in triplicate on YPD plates. Viable yeast counts were performed after incubation for 3 days at 30°C on YNB. All spot tests and viability studies were performed in triplicate.

### Acquired hydrogen peroxide-induced oxidative stress tolerance

Aliquots (50 mL) of yeast cultures were treated with 0.5 mM H_2_O_2_ at 30°C for 1 hr and immediately challenged with 7.5 mM H_2_O_2_. Ten μL aliquots were removed from cultures at 0, 30, and 60 min after the challenge; cells were pelleted and washed before plating on YPD agar. Viability was determined by counting colonies after incubation at 30°C for 24 hrs. Survival was normalised to control samples with control samples defined as being 100% viable.

### Phenotypic microarray analysis

Biolog (Hayward, CA, US) growth medium was prepared using 6 g/L (w/v) glucose, 0.67 g/L (w/v) yeast nitrogen base YNB, 0.2 μL of dye D (Biolog, USA), and supplemented with a nutrient mixture (NSx48- 24 mM Adenine-HCl, 4.8 mM L-histidine HCl monohydrate, 48 mM L-leucine, 24 mM L-lysine-HCl, 12 mM L-methionine, 12 mM L-tryptophan and 14.4 mM uracil). Assays looking at the effect of individual amino acids on hydrogen peroxide-induced stress used amino acids at the same concentration as in the complete NS mixture. For assays using xylose, 6 g/L xylose replaced 6 g/L glucose. Final volume was made up to 30 μl using reverse osmosis (RO) sterile distilled water and aliquoted to individual wells with varying concentrations of appropriate inhibitors.

Strains were prepared for inoculation into the PM assay plates (Biolog, Hayward, CA, US) as follows. Glycerol stocks stored at -80°C were streaked on to YPD plates and incubated at 30°C for approximately 48 h. Two to three colonies from each strain were re-streaked to one section of a fresh YPD plate and incubated overnight at 30°C. Cells were then inoculated into sterile water in 20 × 100 mm test tubes and adjusted to a transmittance of 62% (~5×10^6^ cells/ml).

The cell suspension for inoculation was prepared by mixing 125 μL of cells with 2.65 mL of IFY buffer (Biolog) and adjusted to a final volume of 3 mL by the addition of sterile deionised water. Ninety μL of the above mix was inoculated to each well in a Biolog 96- well plate. Anaerobic conditions were created using Oxygen absorbing packs (Mitsubishi AnaeroPak™System) with an anaerobic indicator (Oxoid, UK) and the plates were placed inside PM gas bags (Biolog). The plates were then placed in the OmniLog reader and incubated for 96 h at 30°C without shaking.

The OmniLog reader (Biology, Hayward, CA) photographs the plates at 15 min intervals, converting the pixel density in each well to a signal value reflecting cell growth and dye conversion. After completion of the run, the signal data was compiled and exported from the Biolog software into Microsoft® Excel for further analysis. In all cases, a minimum of three replicate PM assay runs were conducted, and the average of the signal values was plotted.

### Measurement of yeast growth

Cells were grown to exponential phase in YPD media as monitored by OD_600_ readings, cells were diluted to a starting OD_600_ of 0.2 using YPD broth. H_2_O_2_ (0-5 mM) was added to wells on a 96-well microtitire plate and the volume of each well was adjusted to 100 μL using RO water. Yeast growth (OD_600_) was measured every 15 minutes using a Tecan Infinite M200 Pro plate reader (Mannedorf, Switzerland), at 30°C for 72 hrs. The assay was performed in triplicate and the average reading was plotted.

### Determination of glutathione levels

Glutathione levels, both oxidised and reduced, were determined using a glutathione assay kit (Cayman Chemical Company, US). Samples were prepared for the kit with the cells harvested by centrifugation, washed with phosphate-buffered saline (pH 7.4) to remove any traces of growth medium, and resuspended in ice-cold 8 mM HCl, 1.3% (w/v) 5-sulfosalicyclic acid. Cells were broken with glass beads using a MagNalyser (Roche, Burges Hill, UK) bead beater for 30 s at 4°C, before incubating on ice for 15 min to precipitate proteins. Cell debris and proteins were pelleted in a microcentrifuge for 15 min (17,000 g at 4°C) and the supernatant used for the determination of free glutathione. For quantification of oxidized glutathione (GSSG), samples were pretreated with 5% (v/v) 2-vinyl-pyridine for 1 hr at room temperature before analysis.

### Peroxidase and catalase activity

Peroxidase activity (GPX) was measured using a glutathione peroxidase assay kit (Calbiochem, Merck); glutathione peroxidase activity was determined indirectly by measuring NADPH consumption upon recycling of oxidised glutathione by glutathione reductase. NADPH consumption was measured by a decrease in absorbance (340 nm). Crude cell extracts from cells grown to mid-exponential phase (OD_600_ nm 0.5) were used, cells were harvested and broken in 50 mM Tris-HCl, pH 7.5, 5 mM EDTA and 1 mM dithiothreitol (DTT) using a MagNalyser instrument (Roche Applied Science). Samples were then centrifuged at 13200 g in a microcentrifuge at 4°C to remove unbroken cells. Specific activity was defined as nanomoles of NADPH oxidised per minute per milligram of protein.

Catalase activity (CAT) was measured using a catalase activity kit (Calbiochem, Merck); crude cell extracts from cells grown to mid-exponential phase (OD_600_ nm 0.5) were used, cells were harvested and broken in 50 mM potassium phosphate, pH 7.0 containing 1 mM EDTA using a MagNalyser instrument (Roche Applied Science). Samples were then centrifuged at 13,200 g in a microcentrifuge at 4°C to remove unbroken cells. The assay utilises formaldehyde as a standard for the catalase reactions. Assay buffer containing hydrogen peroxide was added to each well on the plate containing cell-free extracts and incubated on a shaker for 20 minutes. Reactions were stopped by the addition of potassium hydroxide and Purplald. Plates were read at 540 nm. Catalase activity was determined by comparing samples with a formaldehyde reference curve and defined as nmol/min/ml.

## Results

### Sensitive to hydrogen peroxide-induced oxidative stress increased under nutrient deficient growth conditions

**Figure 1:**
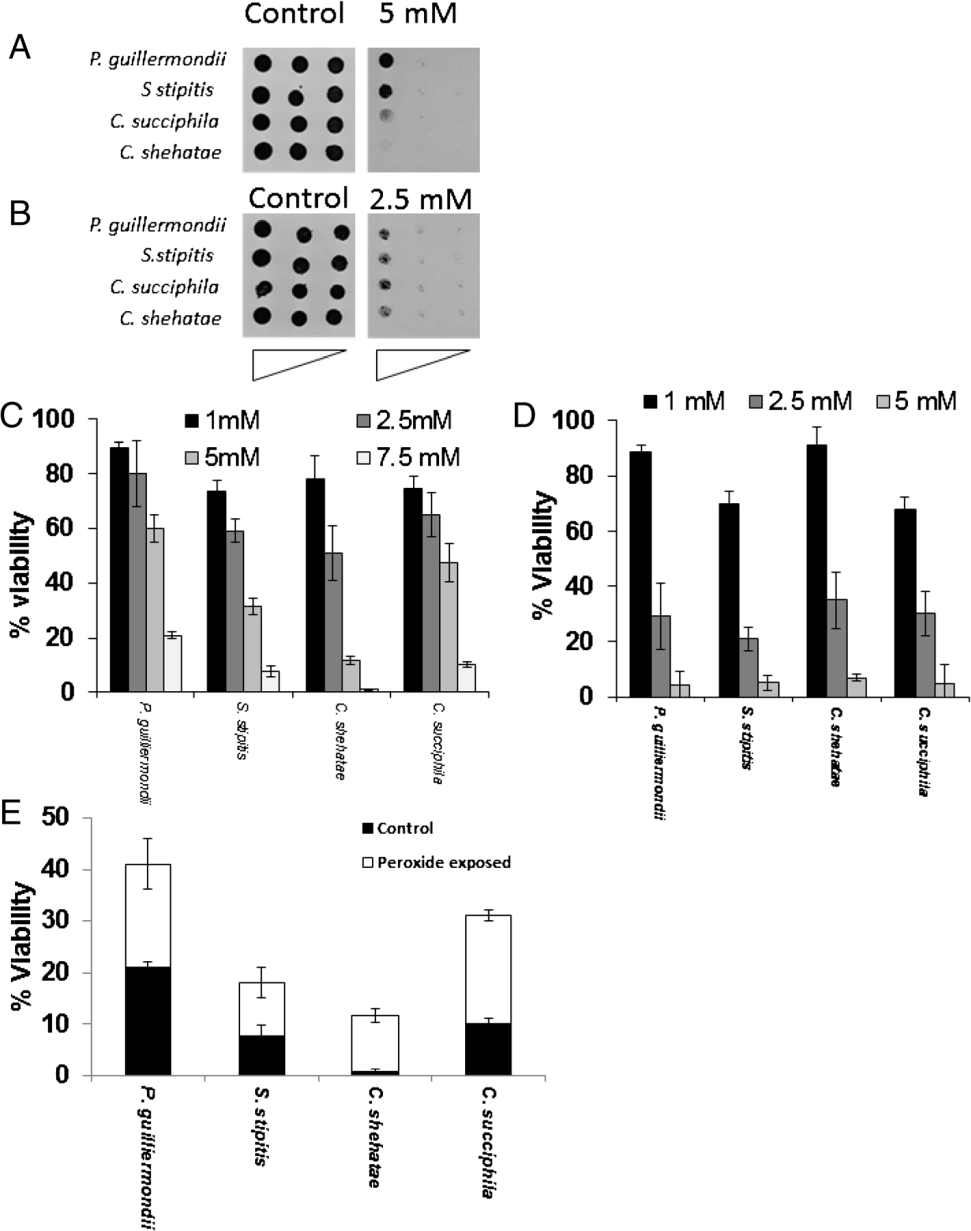
***Candida *and *Pichia *spp are sensitive to hydrogen peroxide-induced oxidative stress. (A)** Cultures of *P. guilliermondii*, *S. stipitis*, *C. succiphila*, and *C. shehatae* were grown to stationary phase and the OD^600^ adjusted to 1.0, 0.1 or 0.01 before strains were spotted onto plates containing various concentrations of hydrogen peroxide. Growth was monitored after 3 days of incubation at 30°C. Results are shown for plates containing no oxidant (YPD) and 5 mM hydrogen peroxide. **(B)** Growth on restrictive media (YNB) makes yeast more sensitive to oxidative stress. Yeast cultures prepared as above were exposed to hydrogen peroxide and grown on restrictive media (YNB). Results are shown for plates containing no oxidant (YNB) and 2.5 mM hydrogen peroxide. **(C)** Yeast spp show a reduced ability to survive oxidative stress. Yeast spp were grown to exponential phase in YPD and treated with 1, 2.5, 5 or 7.5 mM hydrogen peroxide for 1 hour. Percent survival is expressed relative to that of untreated cultures. **(D)** Restrictive growth conditions make yeast more sensitive to oxidative stress. Yeast cultures prepared as above were exposed to hydrogen peroxide in restrictive media (YNB). **(E)** Exposure to hydrogen peroxide stress makes yeast spp hydrogen peroxide tolerant. Yeast spp were maintained under 0.5 mM hydrogen peroxide stress and exposed to 7.5 mM hydrogen peroxide in YPD and viable counts performed as above.

On YPD all yeast in this study were inhibited by 5 mM hydrogen peroxide, 5 mM hydrogen peroxide has been shown to inhibit *Saccharomyces cerevisiae*[31] (Figure 1A). *P. guillermondii* and *S. stipitis* exhibited higher tolerance to hydrogen peroxide-induced oxidative stress when compared with *Candida* spp when grown on YPD containing hydrogen peroxide (Figure 1A). On YNB all yeast in this study exhibited sensitivity to 2.5 mM hydrogen peroxide (Figure 1B). However, viability assays showed that only *P. guillermondii* exhibited higher tolerance to hydrogen peroxide-induced oxidative stress when compared with the other yeast species assayed for in this study (Figure 1C). There was no difference in viability in the presence of hydrogen peroxide when strains were grown in YNB (Figure 1D). Assays with *P. guillermondii* NCYC 441, 3063, *S. stipitis* NCYC 1542 and *C. shehatae* NCYC 3781 confirmed that tolerance to hydrogen peroxide-induced oxidative stress was not within this study strain specific, though further testing on other strains would be desirable (Additional file 1: Figure S1A-S1C).

There was no difference in sensitivity to hydrogen peroxide on YNB between yeast spp (Figure 1D). Yeast pre-incubation with 0.5 mM hydrogen peroxide increased tolerance to 7.5 mM hydrogen peroxide for all yeast in this study (Figure 1E). Increased tolerance to hydrogen peroxide-induced oxidative stress under constant exposure has been reported previously for *S. cerevisiae*[32].

### Hydrogen peroxide-induced oxidative stress inhibits metabolic outout in Candida, Pichia and Scheffersomyces spp

**Figure 2:**
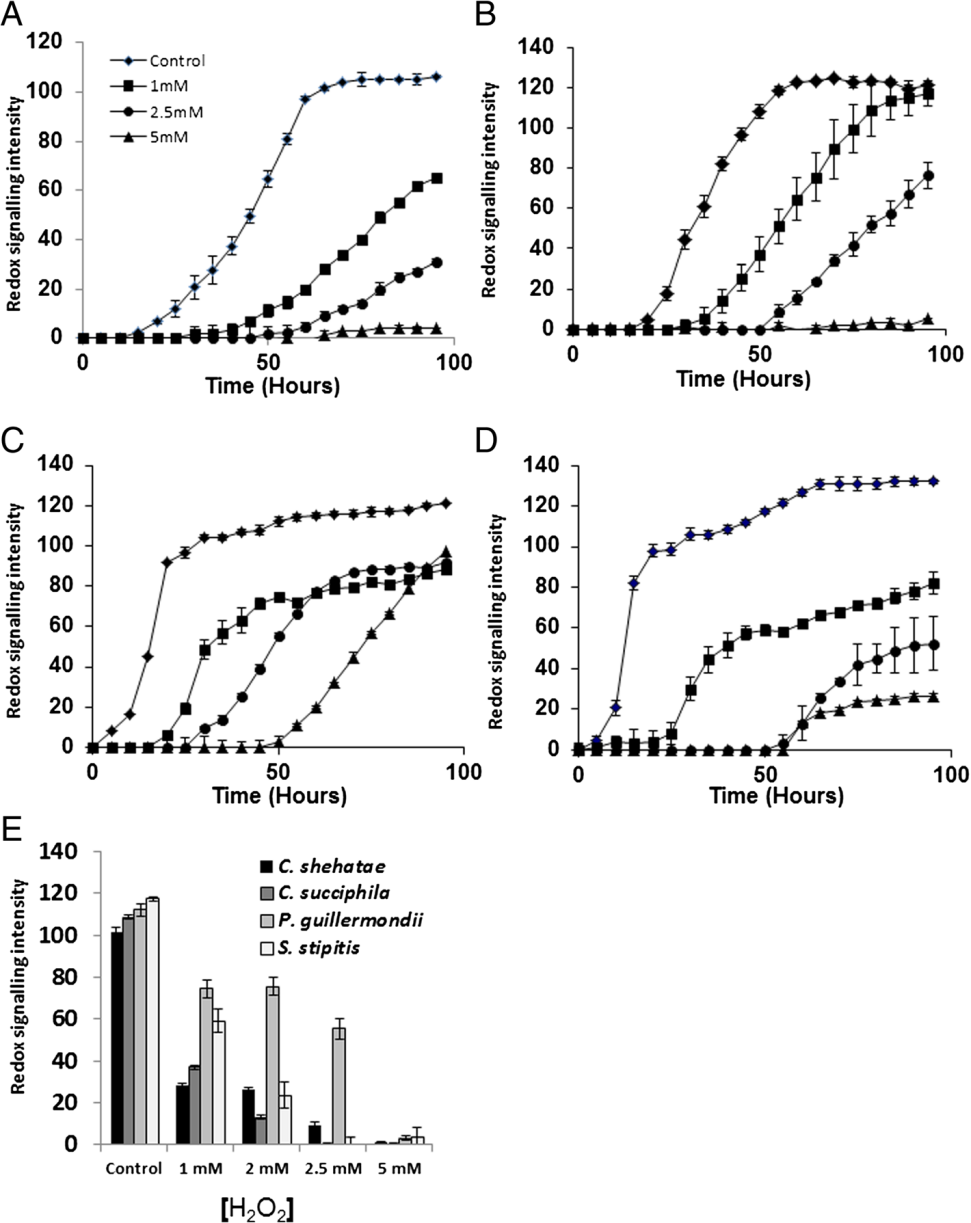
**Oxidative stress prevents metabolic output in yeast spp assayed in this study.** The effect of hydrogen peroxide on metabolic output expressed as redox signal intensity units on yeast spp. **(A)***C. shehatae***(B)***C. succiphila***(C)***P. guilliermondii***(D)***S. stipitis***(E)** Redox signal intensity units for yeast spp under hydrogen peroxide stress after 50 hrs for *C. succiphila*, *C. shehatae*, *P. guillermondii* and *S. stipitis*. The assay was performed in triplicate and the average reading was plotted.

The effect of hydrogen peroxide-induced oxidative stress on metabolic outout was measured using bespoke phenotypic microarray plates for all strains assayed in this study (Figure 2A-D). Presence of hydrogen peroxide was compared with unstressed conditions on yeast metabolic output as measured by a redox sensitive dye. Addition of 5 mM hydrogen peroxide inhibited metabolic output in *Candida* spp (Figure 2A and B) when compared with *P. guillermondii* and *S. stipitis* (Figure 2C and D). We observed that *P. guillermondii* was the most tolerant to hydrogen peroxide-induced oxidative stress when assessed after 50 hours exposure (Figure 2E). This phenotype was confirmed in assays with *P. guillermondii* NCYC 441 in response to hydrogen peroxide-induced oxidative stress (data not shown).

### Hydrogen peroxide-oxidative induced stress inhibits growth in Candida, Pichia and Scheffersomyces spp

**Figure 3:**
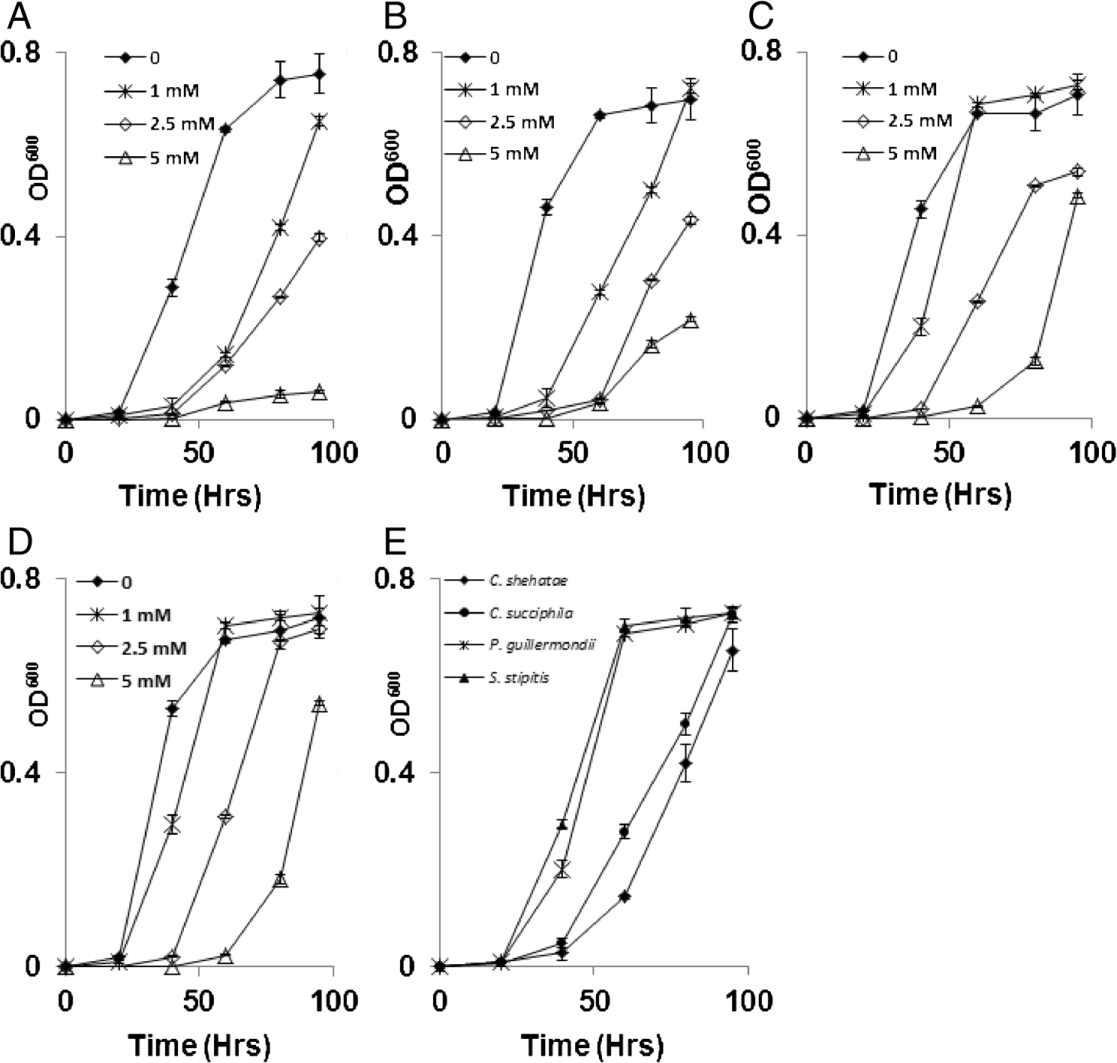
**Effect of hydrogen peroxide on the growth of ethanologenic yeast spp.** Growth in the presence of 0-5 mM hydrogen peroxide was monitored on a TECAN plate reader at OD^600^ for 96 hrs with readings taken every 15 minutes for **(A)***C. shehatae*, **(B)***C. succiphila*, **(C)***P. guillermondii*, and **(D)***S. stipitis* and **(E)** Comparison of inhibition of growth by 1 mM hydrogen peroxide for yeast assayed in this study. The assay was performed in triplicate and the average reading was plotted.

Hydrogen peroxide-induced oxidative stress was characterised by extended lag phases for all yeast assayed in this study (Figure 3A-D). *P. guillermondii* exhibited the highest tolerance to hydrogen peroxide and *C. shehatae* was the least tolerant in terms of growth when compared with other yeast assayed in this study (Figure 3E).

### Addition of amino acids and nucleobases increased tolerance to hydrogen peroxide-induced oxidative stress

**Figure 4:**
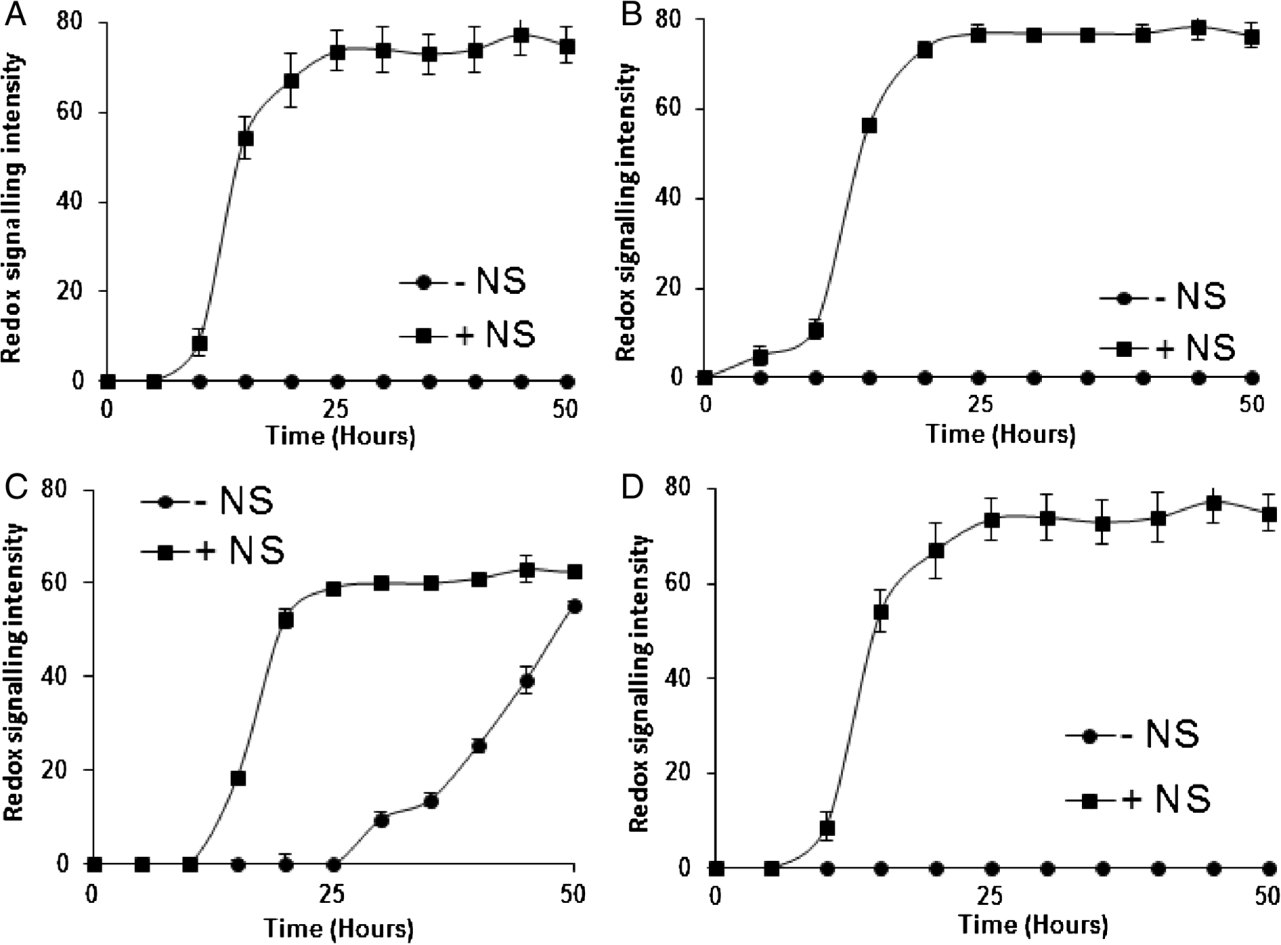
**Effect of nutrient supplementation on hydrogen peroxide sensitivity.** Effect of 2.5 mM hydrogen peroxide was assessed +/- a yeast specific nutrient supplementation containing amino acids on metabolic output, **(A)***C. shehatae*, **(B)***C. succiphila*, **(C)***P. guillermondii*, and **(D)***S. stipitis*. The assay was performed in triplicate and the average reading was plotted.

Yeast strains were more sensitive to hydrogen peroxide-induced oxidative stress when grown on YNB when compared with YPD; Biolog recommend the addition of amino acids and nucelobases for microarray assays with yeast, we investigated the impact of these amino acids and nucleobases (NS) on hydrogen peroxide- induced oxidative stressed yeast. Addition of amino acids and nucleobases elicited an increase in metabolic output in yeast under hydrogen peroxide-induced oxidative stress when compared with assays without amino acids (Figure 4A-D). *P. guillermondii* yeast were observed to be metabolically active under hydrogen peroxide-induced oxidative stress in the absence of NS, however, addition of NS improved tolerance (Figure 4C). We did observe this effect on *P. guillermondii* strain NCYC 441; more observations with strains of *P. guillermondii* are required before this response could be denoted as not strain specific (data not shown). This study was restricted to the amino acids recommended by Biolog, the impact of other amino acids on hydrogen peroxide oxidatively stressed yeast cells was not investigated.

### Addition of adenine and methionine affects metabolic output and cellular growth under hydrogen peroxide-induced oxidative stress

**Figure 5:**
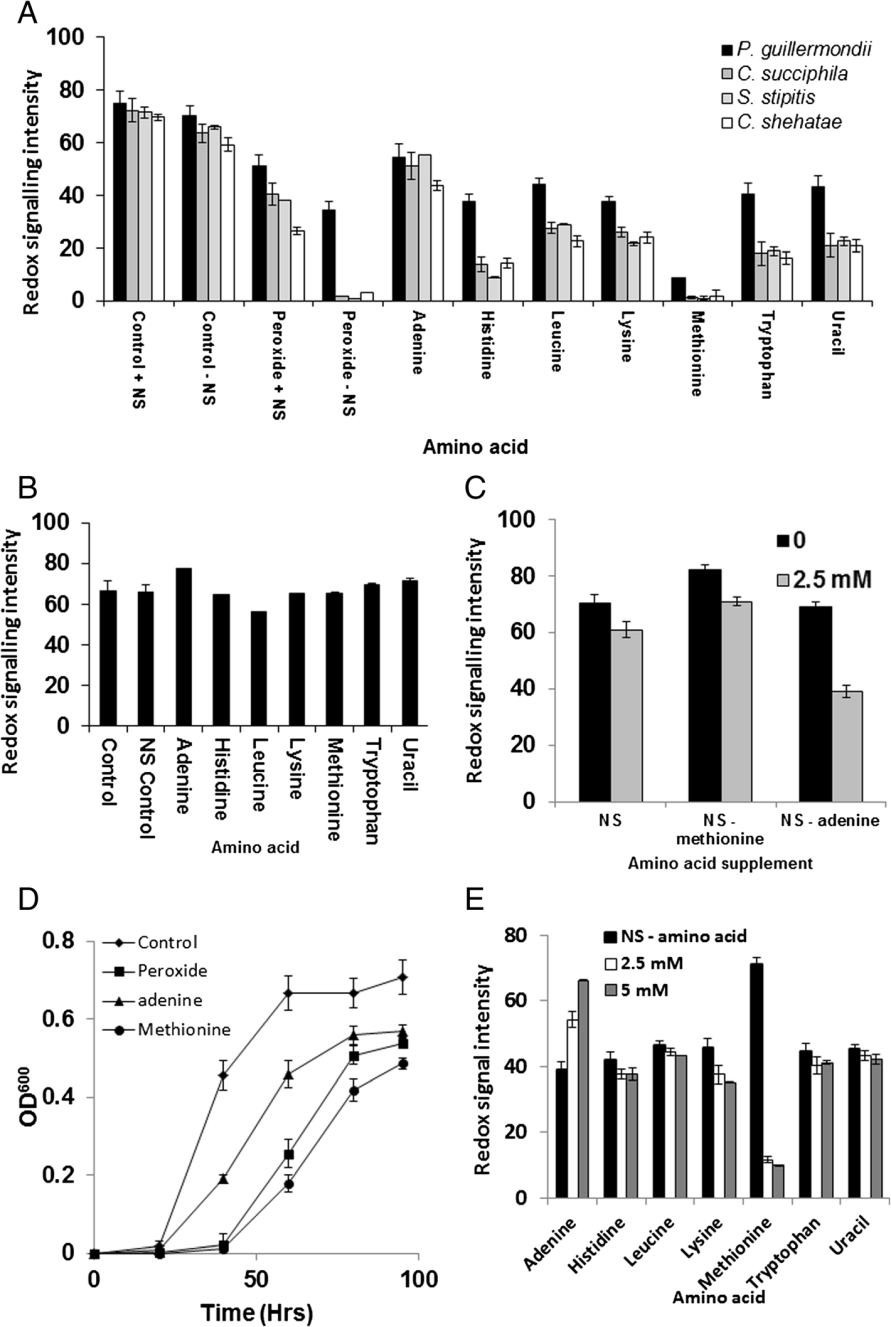
**Effect of individual amino acids on metabolic outout for *P. guillermondii*. (A)** Metabolic output under 2.5 mM hydrogen peroxide stress was measured in the presence of a range of amino acids, **(B)** Effect of amino acids on metabolic output in unstressed yeast cells, **(C)** Effect of NS, NS minus methionine and NS minus adenine on metabolic output under peroxide stress, **(D)** Effect of adenine and methionine on growth under hydrogen peroxide stress and **(E)** Effect of 2.5 mM or 5 mM amino acids on metabolic output of *P. guillermondii* under 2.5 mM hydrogen peroxide-induced oxidative stress. The assays were performed in triplicate and the average reading was plotted.

Addition of adenine (2.4 mM) improved metabolic output compared with controls, presence of leucine, lysine, tryptophan, histidine and uracil also had a slight beneficial effect on observed yeast metabolic output as measured by the conversion of a redox sensitive dye. Addition of methionine had a negative effect on metabolic output for cells under hydrogen peroxide-induced oxidative stress (Figure 5A).

In unstressed cells, addition of amino acids had little effect on metabolic output compared with controls (Figure 5B). Metabolic output was reduced in cells under hydrogen peroxide-induced oxidative stress incubated with methionine and increased with assays using NS minus methionine when compared with NS containing methionine; similarly removal of adenine from NS reduced metabolic output compared with NS containing adenine (Figure 5C). Presence of adenine improved yeast growth when under hydrogen peroxide-induced oxidative stress, whereas addition of methionine was deleterious (Figure 5D). Under control conditions, addition of adenine or methionine had little effect on yeast growth (data not shown). Assays of *P. guillermondii* with 2.5 mM hydrogen peroxide and 2.5 mM and 5 mM of amino acids screened in this study showed that addition of adenine improved metabolic output compared with the other amino acids (Figure 5E). The improvement appeared to be concentration dependent as there was an increase in metabolic output in the presence of 5 mM adenine when compared with assays containing 2.5 mM adenine (Figure 5E).

### The redox state under hydrogen peroxide-induced oxidative stress did not differ between yeast assayed in this study

**Figure 6:**
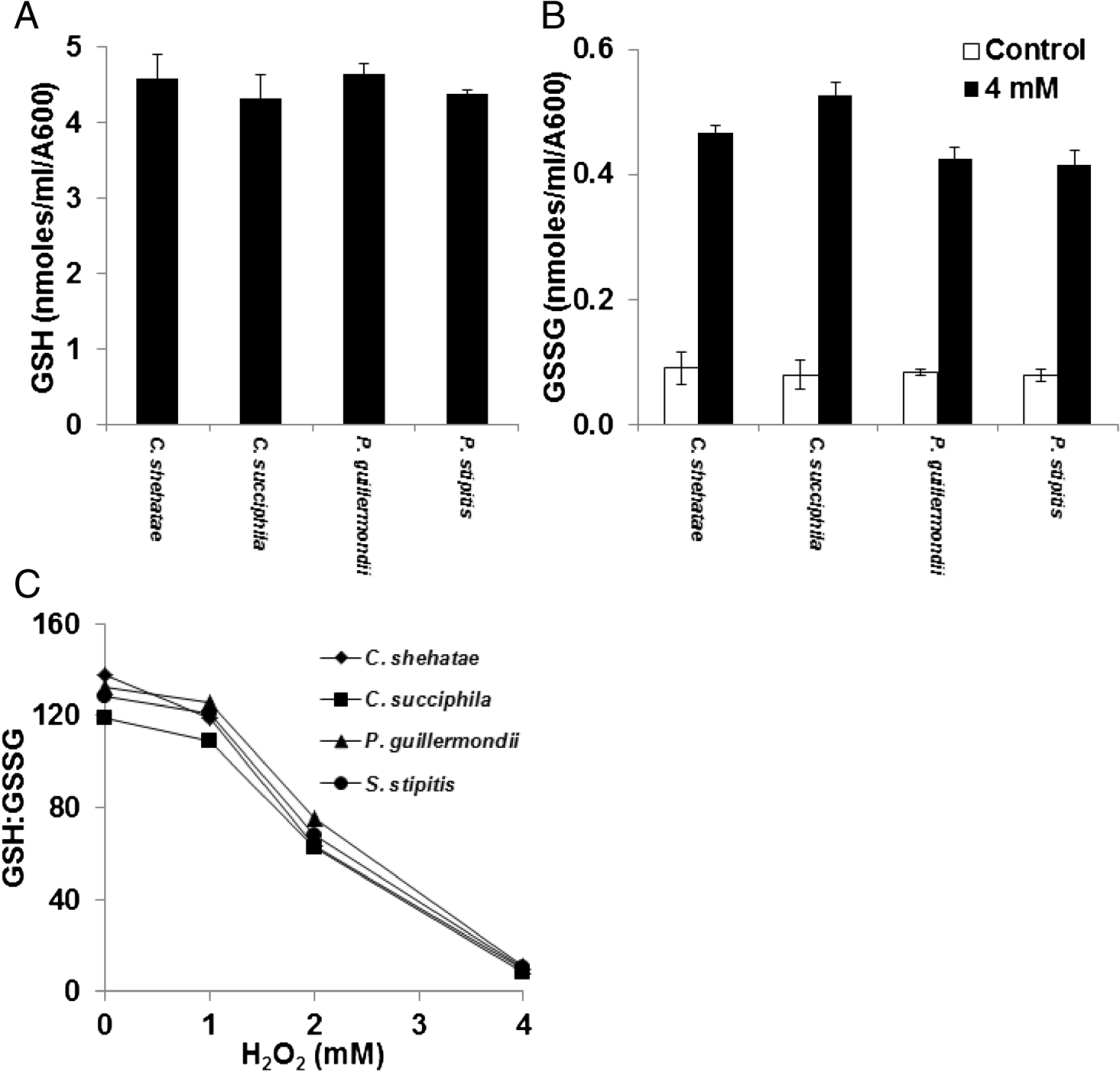
**Effect of hydrogen peroxide-induced oxidative stress on redox status of yeast spp. (A)** Total GSH (nmoles/ml/OD^600^) for yeast spp, **(B)** Effect of hydrogen peroxide-induced oxidative stresson reduced GSSP levels in yeast spp, and **(C)** Effect of hydrogen peroxide-induced oxidative stress on redox balance for yeast spp. The assay was performed in triplicate and the average reading was plotted.

Yeast assayed in this study had total glutathione concentrations of approximately 4.0 nmols/ml/OD (Figure 6A); there were very low levels of oxidised glutathione (GSSG) in unstressed cells. Unstressed cells had a 120-fold ratio of reduced to oxidised glutathione (Figure 6B), under hydrogen peroxide-induced oxidative stress, the ratio shifted to a more oxidised state, this was observed for all yeast assayed in this study (Figure 6C). However, there was little difference between yeast in the redox status under hydrogen peroxide-induced oxidative stress.

### C. shehatae has reduced peroxidase and catalase activity when compared with other yeast assayed in this study

We assayed for peroxidase and catalase activity under hydrogen peroxide-induced oxidative stress for the yeast in this study, along with *S. cerevisiae* NCYC 2592 as a reference strain. *C. shehatae* had significantly lower peroxidase and catalase activity when compared with the other yeast assayed in this study. Catalase activity in *C. succiphila* was lower when compared with other yeast assayed in this study; however, peroxidase activity was not significantly different. However, this response maybe strain specific as other *C. succiphila* strains have yet to be tested for oxidative tolerance or catalase activity. Peroxidase and catalase activity for *S. cerevisiae* is similar to published data for yeast [33,34]. There was no significant difference in peroxidase and catalase activity for *P. guilliermondii* or *S. stipitis* when compared with *S. cerevisiae* (Table 1).

**Table 1:** Peroxidase (GPX) and catalase activity of yeast using either a GPX or catalase assay kit

**Yeast**	**GPX activity nmol/min**^**−1 **^**mg**^**−1**^	**CAT activity nmol/min**^**−1 **^**mg**^**−1**^
*S. cerevisiae*	520.981 ± 2.32	89.154 ± 2.42
*C. shehatae*	494.304 ± 4.23	81.566 ± 3.12
*C. succiphila*	509.383 ± 5.73	84.651 ± 2.63
*P. guillermondii*	513.141 ± 3.01	90.491 ± 1.71
*S. stipitis*	520.415 ± 8.43	88.911 ± 2.53

*C. shehatae* had significantly lower concentrations of peroxidase or catalase activity when compared with other yeast assayed for in this study.

Data expressed as nmol of NADPH oxidised per minute per mg of protein for peroxidase activity and nmol formaldehyde produced in the presence of hydrogen peroxide for catalase activity.

### Utilisation of pentose sugars increases sensitivity to hydrogen peroxide-induced oxidative stress

**Figure 7:**
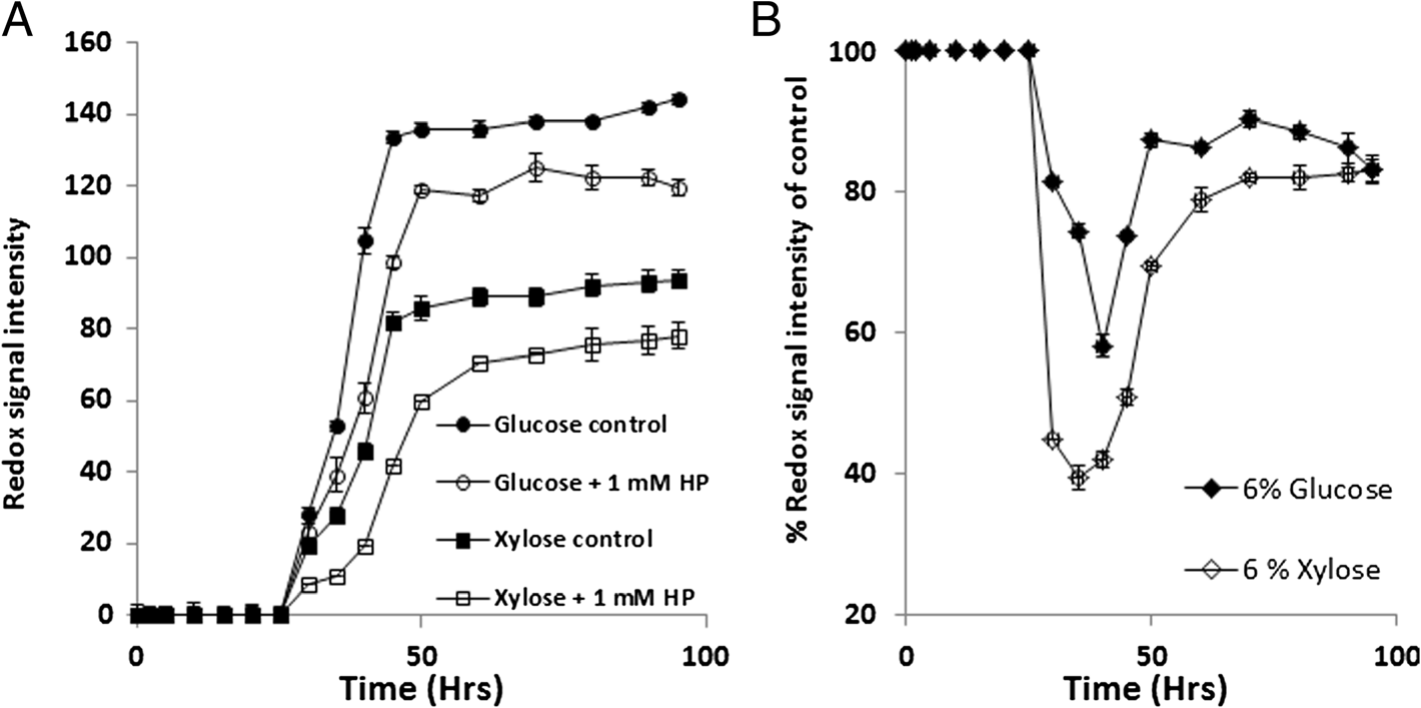
**Effect of altering growth conditions on hydrogen peroxide tolerance for *P. guillermondii*. (A)** Effect of growth on glucose or xylose on hydrogen peroxide tolerance, and **(B)** Effect of carbon source on hydrogen peroxide tolerance expressed as a percentage of unstressed control. The assay was performed in triplicate and the average reading was plotted.

Hydrolysates derived from LCM’s will contain hexose and pentose sugars [35], it has been shown in *S. cerevisiae* that utilisation of xylose induces expression of transcription factors which control response to oxidative stress [36] we measured the impact of hydrogen peroxide-induced oxidative stress on yeast utilising xylose as a sole carbon source. *P. guillermondii* exhibited sensitivity to 1 mM hydrogen peroxide when utilising 6% xylose compared with sensitivity to 5 mM hydrogen peroxide when utilising 6% glucose (Figure 7A). Plotting percentage inhibition of redox signal intensity demonstrated that yeast display greater sensitivity to hydrogen peroxide-induced oxidative stress when utilising xylose compared with glucose (Figure 7B). Increased sensitivity to hydrogen peroxide-induced oxidative stress when utilising xylose was observed for all yeast strains assayed in this study (data not shown).

## Discussion

Utilisation of xylose is an important factor in making second generation biofuels a viable option for the conversion of LCM’s into useable refined products such as ethanol or butanol. Approaches which yield glucose rich and xylose rich streams have been considered for converting all available sugars into high value products. *Candida, Pichia* and *Scheffersomyces* (previously named *Pichia stipitis*) currently represent the best natural yeast for efficient utilisation of pentose sugars [30]. Conversion of pentose sugars such as arabinose into the carotenoid astaxanthin by the yeast *Phaffia rhodozyma* rather than conversion of ethanol in poor ethanol producing yeast is of particular interest to the bioethanol industry [37]. Oxidative stress induced by free radical production by metabolising cells is a consequence of the industrial fermentation processes, where yeast are serially sedimented out of the media and reused in subsequent fermentations, during these stages yeast are exposed to oxygen, and oxidative stress has been observed to reduce viability, induce glycerol accumulation and reduce ethanol production [38].

We assayed for the tolerance to hydrogen peroxide-induced oxidative stress in pentose utilising yeast using a be-spoke phenotypic microarray plates in which the effect of hydrogen peroxide can be compared against unstressed conditions. We measured the effect of hydrogen peroxide and assessed when each yeast was affected (i.e., an extended lag phase when compared with control) and when complete inhibition of metabolic output was observed. We confirmed the data using traditional spot plates, viability and effect on growth using a plate reader.

All yeast assayed in this study exhibited a similar tolerance to hydrogen peroxide-induced oxidative stress as those reported for *S. cerevisiae*[31]. However, *P. guillermondii* was more tolerant to hydrogen peroxide-induced oxidative stress when compared to *C. shehatae, C. succiphila and S. stipitis* based on metabolic output, growth and viability assays. This tolerance appeared to be yeast specific rather than a characteristic of that strain of the yeast as assays with other *P. guillermondii* confirmed the phenotypic response to hydrogen peroxide-induced oxidative stress. Though strain variation in response to an oxidant is always possible in any population, variation in response has been observed with the same strain when obtained from different geographic sources [39,40].

All of the yeasts were more sensitive to hydrogen peroxide when grown on YNB as opposed to YPD, however, addition of adenine improved tolerance to hydrogen peroxide-induced oxidative stress. Yeast incubated with NS minus adenine were more sensitive to hydrogen peroxide-induced oxidative stress when compared with NS containing adenine, and incubation with NS minus methionine improved oxidative tolerance compared with NS containing methionine. Adenine has been shown to induce adenosine triphosphate (ATP) production, ATP has been shown to buffer cells against oxidative stress, and is required for the activity of a mitochondrial transporter which is essential for yeast cells undergoing oxidative phosphorylation, additionally adenine is a key component of co-factors and enhancing NADPH supply has been suggested as improving xylose fermentations [41-43]. We have yet to determine the precise nature of the protective role of adenine on hydrogen peroxide-induced oxidative stressed yeast.

Exposure to high concentrations of methionine has been shown to induce apoptotic markers and lipid peroxidation in rat liver cells [44], methionine also induces ROS in rat liver mitochondria with associated mitochondria DNA damage [45]. However, yeast such as *S. cerevisiae* does not require polyunsaturated fatty acids that are the primary targets of such peroxidation induced by ROS and normally contain mainly saturated or monounsaturated fatty acids [46]. An adverse effect of methionine on yeast cells under hydrogen peroxide-induced oxidative stress has not been reported previously.

Glutathione (GSH) concentrations in *Candida*, *Pichia and Scheffersomyces* cells were found to be similar to levels quoted for exponentially growing *S. cerevisiae* cells [24], there was no difference in GSH levels between the yeast spp. Hydrogen peroxide-induced oxidative stressed cells are characterised by an increase in oxidised glutathione (GSSG) and a shift in redox balance. There was no difference in GSSG concentrations or redox balance between the yeast spp assayed in this study. The increased sensitivity of *Candida* spp to hydrogen peroxide is not due to differences in the glutathione maintained redox balance of the cell.

Peroxidase and catalase activity in *C. shehatae* was significantly reduced when compared with other yeast assayed in this study. Catalase activity for *C. shehatae* and *C. succiphila* was significantly reduced when compared with *S. cerevisiae*, *P. guillermondii* or *S. stipitis*. All yeast assayed in this study exhibited increased sensitivity to hydrogen peroxide-induced oxidative stress when utilising xylose as a sole carbon source when compared with glucose. Hydrolysates from LCM’s will contain variable concentrations of glucose and pentose sugars, thus this study suggests that yeast metabolising pentose sugars will be more sensitive to oxidative stress compared to those metabolising hexose sugars.

## Conclusions

This study has determined that *Candida*, *Pichia and Scheffersomyces* exhibit tolerance to hydrogen peroxide-induced oxidative stress at concentrations (1- 5 mM) similar to *S. cerevisiae*. However, *P. guillermondii* was more tolerant to hydrogen peroxide-induced oxidative stress when compared to *C. shehatae, C. succiphila or S. stipitis* based on metabolic output, growth and viability assays. We failed to observe differences in the redox status of the yeast under hydrogen peroxide-induced oxidative stress but there were differences in key antioxidant response enzymes to hydrogen peroxide-induced oxidative stress. We observed that addition of adenine improved tolerance to hydrogen peroxide-induced oxidative stress whereas addition of methionine was deleterious.

## Competing interests

The authors declare that they have no competing interests.

## Authors’ contributions

JS: Performed the phenotypic microarray experiments, viability and growth assessments. TGP: Helped with the writing of this manuscript and helped guide the research. KAS: Helped with the writing of this manuscript and helped guide the research. DG: Performed the glutathione/GSSG assays, catalase + peroxidase assays, wrote the paper and guided JS’s research. All authors read and approved the final manuscript.

## Supplementary Material

Additional file 1: Figure S1 Oxidative stress prevents metabolic outout in yeast spp assayed in this study. The effect of hydrogen peroxide on metabolic output expressed as redox signal intensity units on yeast spp. **(A)***P. guillermondii* NCYC 441 and NCYC 443 **(B)***S. stipitis* NCYC 1541 and NCYC 1542 **(C)***C. shehatae* NCYC 2389 and NCYC 3781 The assay was performed in triplicate and the average reading was plotted. Click here for file
