# Supplementary material for: Tolerance of pentose utilising yeast to hydrogen peroxide-induced oxidative stress
Source: BMC Res Notes. 2014 Mar 17;7:151. doi: 10.1186/1756-0500-7-151 (PMC4004043; doi:10.1186/1756-0500-7-151)
Supplement: Additional file 1: Figure S1 — Oxidative stress prevents metabolic outout in yeast spp assayed in this study. The effect of hydrogen peroxide on metabolic output expressed as redox signal intensity units on yeast spp. (A)P. guillermondii NCYC 441 and NCYC 443 (B)S. stipitis NCYC 1541 and NCYC 1542 (C)C. shehatae NCYC 2389 and NCYC 3781 The assay was performed in triplicate and the average reading was plotted. [file 1756-0500-7-151-S1.pptx]

## Slide 1
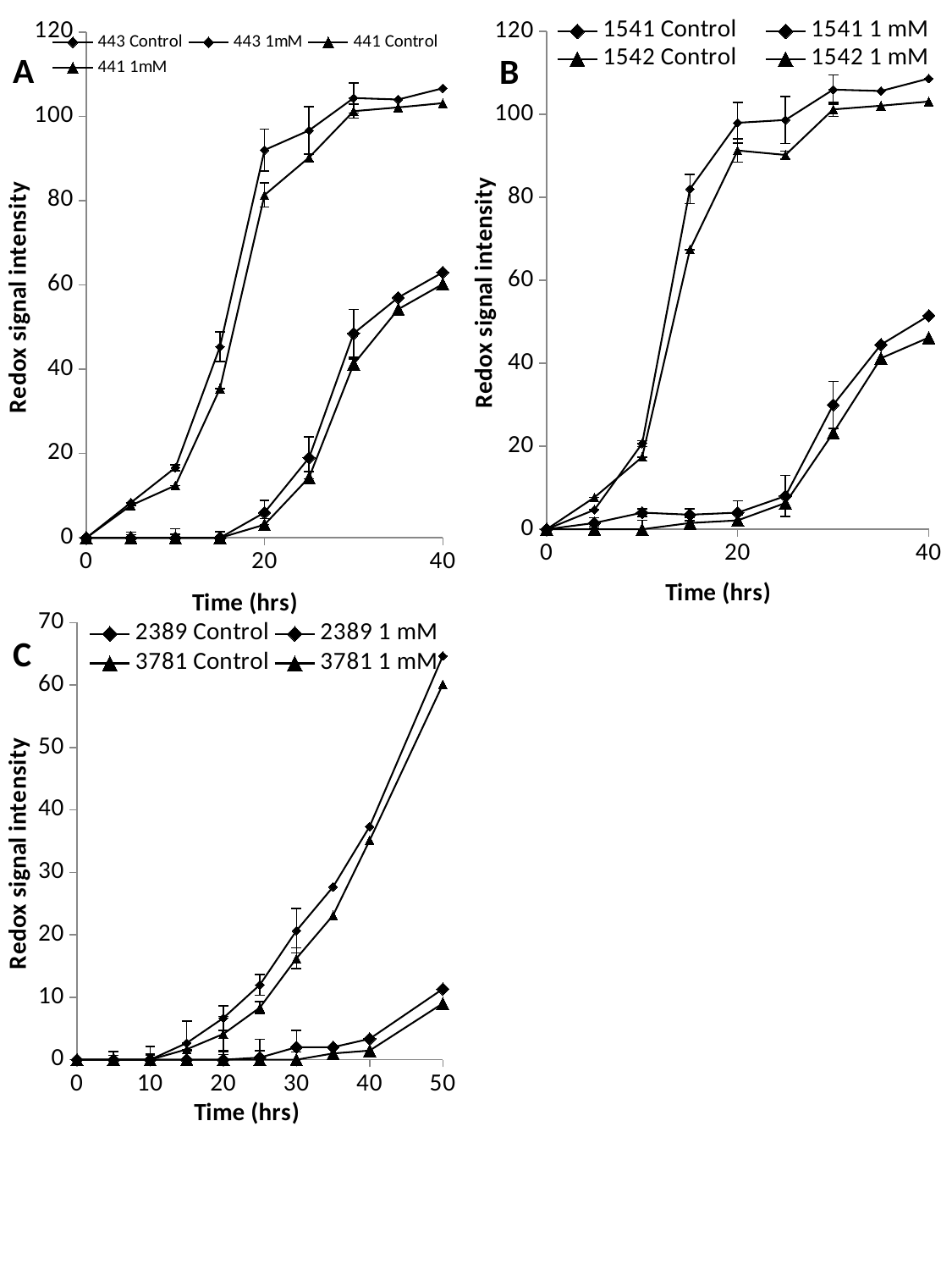

### Chart
| Category | 443 Control | 443 1mM | 441 Control | 441 1mM |
|---|---|---|---|---|
### Chart
| Category | 1541 Control | 1541 1 mM | 1542 Control | 1542 1 mM |
|---|---|---|---|---|A
B
### Chart
| Category | 2389 Control | 2389 1 mM | 3781 Control | 3781 1 mM |
|---|---|---|---|---|C
